# Gene Cloning, Characterization, and Molecular Simulations of a Novel Recombinant Chitinase from Chitinibacter Tainanensis CT01 Appropriate for Chitin Enzymatic Hydrolysis

**DOI:** 10.3390/polym12081648

**Published:** 2020-07-24

**Authors:** Yeng-Tseng Wang, Po-Long Wu

**Affiliations:** 1 Department of Biochemistry, College of Medicine, Kaohsiung Medical University, Kaohsiung 80708, Taiwan; 2 Drug Development and Value Creation Research Center, Kaohsiung Medical University, Kaohsiung 80708, Taiwan; 3 Graduate Institute of Medicine, Kaohsiung Medical University, Kaohsiung 80708, Taiwan; 4 Department of Medical Research, Kaohsiung Medical University Hospital, Kaohsiung 80708, Taiwan; 5 School of Post-Baccalaureate Medicine, College of Medicine, Kaohsiung Medical University, Kaohsiung 80708, Taiwan; 6 Biotech Business Center, Refining & Manufaturing Research Institute, CPC Corporation, Chiayi City 60051, Taiwan; 078760@cpc.com.tw

**Keywords:** chitinase, gene cloning, bioassay, molecular simulations

## Abstract

Chitin, a polymer of N-acetyl-d-glucosamine (GlcNAc), can be degraded by chitinase, which is produced by higher plants, vertebrates, and bacteria. Chitinases are characterized by the ability to hydrolyze the beta-1,4-linkages in the chitin chain by either an endolytic or an exolytic mechanism. Chitinase 1198 is a novel endochitinase from the genome sequence of *Chitinibacter tainanensis* CT01. Herein, we report the findings of molecular simulations and bioassays for chitinase 1198. Our experimental results suggest that chitinase 1198 can recognize the nonreducing end of chitin and cleave the second or third glycosidic linkage from the nonreducing end of chitin oligomers. Furthermore, our simulations results revealed that chitinase 1198 is more likely to bind chitin oligomers with the main hydrogen bonds of the Asp440, the second GlcNAc unit of chitin oligomers, and degrade chitin oligomers to (GlcNAc)_2_ molecules. Moreover, chitinase 1198 is less likely to bind chitin oligomers with the main hydrogen bonds of the Asp440, the third GlcNAc unit of chitin oligomers, and degrade chitin oligomers to (GlcNAc)_3_ molecules. Lastly, chitinase 1198 can bind (GlcNAc)_3_ molecules with the main hydrogen bonds of the Asp440, the second GlcNAc of the (GlcNAc)_3_ molecules, and degrade chitin oligomers to GlcNAc and (GlcNAc)_2_ molecules.

## 1. Introduction

Chitins have been used as functional materials in the food and health fields because of their biocompatibility and nontoxicity. The poor solubility and high molecular weight of chitin polymers limit their potential use; however, this problem can be overcome by using their derived oligomers and monomers [1]. The production of soluble chitin oligomers and monomers is based on a hydrolysis of acetamide group in marine polysaccharides, and there are chemical and enzymatic methods to produce the soluble chitin oligomers and monomers. The advantages of chemical methods include a short time processing time and applicability at the industrial scale. Chemical deacetylation has many disadvantages, such as high energy consumption, environmental pollution, and a decrease in chitin’s mechanical properties. The enzymatic hydrolysis has several advantages over the traditional chemical methods, such as avoiding the environmental pollution problem and decrease in the chitin mechanical properties, while producing chitin with suitable molecular weight and the desired degree of deacetylation [2]. 

Family 18 chitinases are broadly distributed in a variety of organisms, such as plants, bacteria, fungi, mammals, and viruses [3]. Class I chitinases are found in plants, whereas class II enzymes are contained in plants, fungi, and bacteria [4]. Chitinases are divided into two main categories, namely endochitinase (EC 3.2.1.14) and exochitinase (EC 3.2.1.29) [5,6,7,8,9]. Endochitinases randomly hydrolyze chitin at internal sites to produce chitotriose, chitotetraose, and diacetylchitobiose. Exochitinases have two subcategories, namely chitobiosidases and β-1, 4 N-acetyl glucosaminidases. Chitobiosidases (EC 3.2.1.29) can catalyze the progressive release of diacetylchitobiose starting at the nonreducing end of chitin. 1-4-β-glucosaminidases (EC 3.2.1.30) can cleave the oligomeric products of endochitinases and chitobiosidases, generating monomers of GlcNAc. To produce soluble GlcNAG monomers and oligomers from chitin polymers, the highly efficient chitinases are thus desirable and urgently required.

A novel bacterial species, *Chitinibacter tainanensis* CT01, has been found and isolated in Taiwan. *C. tainanensis* CT01 exhibits a significantly higher efficiency in hydrolyzing chitin polymer to GlcNAG oligomers and monomers. The genome of *C. tainanensis* CT01 has been sequenced and analyzed. The genomic information revealed that 12 protein-coding genes might function as endochitinases. In total, 10 chitinase-encoding genes belong to GH18 (glycosyl hydrolase, family 18), and 2 belong to GH19 (glycosyl hydrolase, family 19). To understand the evolutionary relationship of these 12 chitinase-encoding genes, Blast software was used to build their phylogenetic tree and those of other chitinase gene sequences (Figure S1). Data revealed that our 12 chitinase-encoding genes can be classified into 10 groups with low sequence similarities, and the chitinase-encoding genes are isoenzymes (Figure S1). The 12 chitinase-encoding genes were successfully cloned and overexpressed for screening potential endochitinase candidates by using pNP-(GlcNAc)_3_ (p-Nitrophenyl beta-glycosides of N-acetylchitooligosaccharides) as a substrate. Our preliminary results indicated that chitinase 1198 (CT01GL001198 in Figure S1) had the highest chitinolytic activity toward the pNP-(GlcNAc)_3_ substrate. Therefore, chitinase 1198 was used to study the kinetic and catalyst function.

Three-dimensional (3D) structural information is essential to understand an enzyme’s kinetic and catalyst functions. However, the 3D structure of chitinase 1198 has not been completely characterized. Molecular simulation techniques have been successfully applied to study the interactions of chitinase with their substrates [10,11,12,13,14]. Thus, homology modeling techniques were used to generate a 3D structure of chitinase 1198 in the absence of an X-ray crystal structure [15]. Molecular docking and molecular dynamics (MD) simulations were used to generate the initial 3D structure of chitinase 1198 with chitin oligomers. In this study, the function and 3D structure of chitinase 1198 were determined using molecular biology, enzyme kinetics, and molecular simulations. 

## 2. Materials and Methods 

p-Nitrophenyl-(N-acetyl-b-D-glucosaminide)1–3 [p-NP-(GlcNAc)1–3] compounds were obtained from Sigma Chemical Co. (St. Louis, MO, USA), and chitin polymers were obtained from Charming & Beauty Co. (Taipei, Taiwan). 

### 2.1. Plasmid Construction 

The expression plasmid for chitinase 1198 was constructed using the polymerase chain reaction as follows. Two oligonucleotides, sense (5′-CACCATGTCGCACTTTAATCGTTT TGC-3′) and antisense (5′-CTGACGGGCTTTACCCATG-3′), and *C. tainanensis* genomic DNA were used as primers and a template for DNA amplification, respectively. Amplified DNA was digested using a restriction enzyme and then ligated with a vector pET-101/D-TOPO. DNA sequencing confirmed no mutation in the sequence of the insert.

### 2.2. Preparation of Colloidal Chitin

Colloidal chitin was prepared by the method of Murthy and Bleakley [16,17]. A total of 5 g chitin powder was treated with 50.0 mL of concentrated hydrochloric acid (HCl) in an ice bath. Chilled HCl was incorporated gradually with continuous stirring until a thick paste was formed. For equilibration purposes, this slurry was stirred (150 rpm) for 60.0 min in an ice bath. Two liters of ice-chilled distilled water was incorporated with constant stirring for the formation of colloidal chitin precipitates. This suspension was further kept at 4.0 °C overnight. The precipitates were washed extensively until a neutral pH was obtained. Then, we measured the moisture content of colloidal chitin by moisture meters. Colloidal chitin was autoclaved at 121.0 °C for 15.0 min and was stored at 4.0 °C for further chitinase 1198 enzymatic kinetic analysis.

### 2.3. Culture Conditions and Protein Purification 

*Escherichia coli* DH5 alpha strain and *E. coli* BL21 (DE3) strain were used for plasmid construction and heterologous gene expression, respectively. *E. coli* cells were cultivated at 37 °C in Luria–Bertani (LB) medium containing ampicillin (0.1 mg/mL) at 25 °C with vigorous aeration overnight. A fresh overnight culture was used to inoculate 1 L of the LB medium until optical density at 600 nm reached approximately 0.4. Isopropyl-β-D-thiogalactopyranoside (IPTG) was added to a final concentration of 0.5 mM to induce overexpression. After cells were cultivated for a further 7 h, they were harvested through centrifugation at 5000× *g* for 20 min at 4 °C. Cell pellets were resuspended with 50 mM Tris-HCl (pH = 7.5) supplemented with 0.2 mg/mL of lysozyme, 50 U/mL of benzonase, and 0.0005 mg/mL of a cocktail protease inhibitor. These cell suspensions were disrupted using sonication, and crude cell extracts were obtained through centrifugation at 5000× *g* for 20 min at 4 °C. Supernatants after centrifugation were applied to an Ni column, followed by gel-filtration chromatography on a Superose 12 30/300 GL (GE Healthcare Biosciences), and equilibrated with 50 mM Tris-HCl (pH = 7.5). Peak fractions obtained after applying a gel filtration column were collected and analyzed using sodium dodecyl sulfate-polyacrylamide gel electrophoresis (SDS-PAGE) according to Laemmli’s method [18]. Sodium dodecyl sulfate polyacrylamide electrophoresis (SDS–PAGE) was carried out in 10.0% polyacrylamide gel. PageRuler^TM^ prestained protein ladder—(Thermo Scientific) were used. An electrolysis buffer of tris-tricine-0.1% (*w*/*v*) SDS was used with 10% to 20% polyacrylamide gradient gel. The proteins in the gel were visualized by Coomassie Brilliant Blue R-250 staining.

### 2.4. Effects of pH and Temperature on Chitinase Activity 

Chitinase activity was measured under various pH and temperature conditions [19]. The optimum temperature for chitinase 1198 activity was determined by incubating enzyme preparations with p-NP-(GlcNAc)_2_ substrate at various temperatures between 30 and 90 °C at pH 7.0. Briefly, 21-μL protein samples (0.153 M) were added into 300 μL of 0.18 mM p-NP-(GlcNAc)_2_ in 50 mM Tris-HCl buffer (pH = 7.0) and incubated at different temperatures for 1 h. The reaction was ended by adding 10 μL of 1 M NaOH, and the amount of p-nitrophenol released from p-NP-(GlcNAc)_2_ was measured by recording absorbance at 405 nm [20]. To determine the thermostability of chitinase 1198 activity, 21 μL of protein samples (0.153M) were preincubated at different temperatures (20 °C to 90 °C) without p-NP-(GlcNAc)2 substrate for 1 h. After preincubation, protein samples were placed in ice for 30 min. Then, these samples were followed by the addition of 300 μL of 0.18 mM p-NP-(GlcNAc)2 to 50 mM Tris-HCl buffer (pH = 7.0) and then incubated for another 1 h [20]. Moreover, the activity of the non-heated enzyme was defined as 100%. The response of chitinase to pH was determined using the aforementioned standard assay but with the addition of different buffer systems appropriate for the pH under investigation. All buffers had a concentration of 50 mM, and buffer systems and pH ranges employed were as follows: sodium citrate (pH = 2.0–5.0), sodium phosphate (pH = 5.0–6.0), MES buffer (pH = 6.0–7.0, 2-(N-morpholino)ethanesulfonic acid), Tris-HCl (pH = 7.0–9.0), and glycine-NaOH (pH = 9.0–12.0). The effect of metal ions was evaluated using the same methods in the presence of 10 mM different ions. Enzyme activity without the addition of metal ions was measured simultaneously, and the relative activities in the presence of metals were calculated. All chitinase activity values were taken as the means of three replications. To determine exochitinase and endochitinase activity, p-NP-(GlcNAc)_1–3_ was separately used as a substrate in the standard chitinase activity assay. One unit of chitinase activity was defined as the release of 1 μmoL p-nitrophenol per minute. Kinetic constants for chitinase were determined using the standard assay but varying the p-NP-(GlcNAc)_2_ concentration between 5 and 300 μM. The Michaelis–Menten constant (*K*_M_) and turnover number (*k*cat) were determined using hyperbolic regression with Prism software (https://www.graphpad.com/scientific-software/prism/) [21]. 

### 2.5. Chitinase Assay Using Thin-Layer Chromatography 

Reaction products for various N-acetyl-chitooligosaccharides and colloidal chitin were analyzed using silica gel thin-layer chromatography (TLC). Aliquots (10 L) of reaction mixturesysis was carried according to the method of Tanaka et al. [22]. Aliquots (10 L) of reaction mixtures were spotted twice on TLC plates (TLC Silica gel 60 F254, Merck, Taipei, Taiwan) using micro-capillary (Drummond, Scientific, Broomall, Pennsylvania, USA), along with the respective (GlcNAG)_1–3_ standards. The plates were air dried. The mobile phase (the n-butanol:methanol:25% of ammonia:water ratio was 5:4:2:1) was allowed to run along the TLC plate until the solvent front reached more than 3/4 of its length, after which the plate was marked and dried. Then, the plates were developed by spraying with the reagent (4 mL of aniline, 4 g of diphenylamine, 200 mL of acetone, and 30 mL of 85% phosphoric acid) and heated at 180 °C for 3 min. Then, the TLC image was photographed using a Nikon Coolpix L110 digital camera (Nikon, Tokyo, Japan)

### 2.6. Effects of Metal Ions and Coumpound on Enzyme Activity 

The enzyme was added to a final concentration of 1 mM in the substrate of different metal ions such as (K^+^, Na^+^, Ba^2+^, Ca^2+^, Co^2+^, Cu^2+^, Mn^2+^, Mg^2+^, and Zn^2+^) and chemical compound (EDTA). Then, they were incubated at 40 °C for 1 h. The relative activity of the treated enzyme to that of the untreated one was explained by the percentage ratio.

### 2.7. Molecular Modeling of Chitinase 1198

The I-TASSER homology program package [23] was used to model chitinase 1198 protein. To obtain a reasonable number of protein conformations, predicting models were subjected to an energy minimization process by using the conjugate gradient method for approximately 2000 iterations and 10 ns of isothermal constant-volume MD simulation with AMBER FF99SB all-hydrogen amino acid parameters in the AMBER18 (pmemd.cuda) program [24]. To assess the quality of these prediction models, ERRAT [25] analysis was also performed. We considered two aspects in choosing the model, namely the ERRAT quality factor (approximately 94.36) and the optimum binding site for accommodating chitin oligomers. Subsequently, chitin oligomers (GlcNAc repeat unit: 12 and 3) were constructed and minimized using a carbohydrate builder. The AutoDock Vina program was used to dock chitin oligomers into the active site of the model structure (family 18 chitinase: DXXDXDXE one letter amino acid motif) [26,27]. A total of 100 conformations were obtained from docking and then scored. Binding modes were identified after sorting by the AutoDock score and comparing the 100 conformations with the chitin oligomers of chitinase 1198. The conformations with the highest AutoDock score and similarity with the chitin oligomers of chitinase 1198 were selected for next simulations. For chitinase 1198 with chitin oligomers (GlcNAc repeat unit: 12), the distance between the Asp 440 of chitinase 1198 and the nitrogen atom of the second GlcNAc repeat unit from the nonreducing end was constrained within 3.0 Å and minimized for 10,000 conjugate gradient steps by using Tripos Sybyl software. The same was performed for the distance between the Asp 440 of chitinase 1198 and the nitrogen atom of the third GlcNAc repeat unit. For chitinase 1198 with chitin oligomers (GlcNAc repeat unit: 3), the distance between the Asp 440 of chitinase 1198 and the nitrogen atom of the second GlcNAc repeat unit from the nonreducing end was constrained within 3.0 Å and minimized for 10,000 conjugate gradient steps by using Tripos Sybyl software. Then, these complex structures were generated and inserted into TIP3P solvent molecules. The size of the complex structures was approximately 11.62 × 12.42 × 8.37 nm^3^. Then, these initial complexes were simulated using the AMBER 18 package with the AMBER FF99SB all-hydrogen amino acid [28,29,30]. All MD simulations were performed in the isothermal–isobaric (NPT) assembly with a simulation temperature of 310 K, unless stated otherwise, by using the Verlet integrator with an integration time step of 0.002 ps and SHAKE constraints [31] for all covalent bonds involving hydrogen atoms. In electrostatic interactions, atom-based truncation was performed using the PME [32] method, and the switch van der Waals function was used with a 2.00-nm cut-off for atom–pair lists. These complex structures were minimized for 100,000 conjugate gradient steps and then subjected to 1-ns NVT and 100-ns NPT MD simulations. Complex structures from the 100-ns NPT MD simulations were used for solvated interaction energy (SIE) free energy calculations and radial distribution function (RDF) analysis by using cpptraj software [33].

### 2.8. SIE Free Energy Calculations 

The binding free energies of these systems were calculated for snapshot structures. The SIE [34] function is expressed as follows:

(1) $$\Delta G_{bnd}\left(\rho ,D_{in},\;\alpha ,\gamma ,C\right)=\alpha \times \left[E_{c}\left(D_{in}\right)+\Delta G_{bind}^{R}\left(\rho ,D_{in}\right)+E_{vdw}+\gamma \Delta MSA\left(\rho \right)\right]+C$$

where $E_{C}$ and $E_{vdw}$ are the intermolecular Coulomb and van der Waals interaction energies, respectively. The two values were obtained using the AMBER force field (FF99SB). The term $\Delta G_{bind}^{R}$, estimated by solving the Poisson equation, refers to the reaction field energy change between the free and bound states. BRI and BEM [35,36] are molecular surfaces generated using a variable-radius solvent probe [37]. Δ*MSA* refers to the change in the molecular surface area upon binding. The constant values of these parameters are *α* = 0.1048, *Din* = 2.25, *ρ* = 1.1, γ = 0.0129 kcal/(mol Å^2^), and *C* = −2.89 kcal/mol [38]. 

## 3. Results

### 3.1. Overexpression and Purification of Chitinase 1198

**Figure 1:**
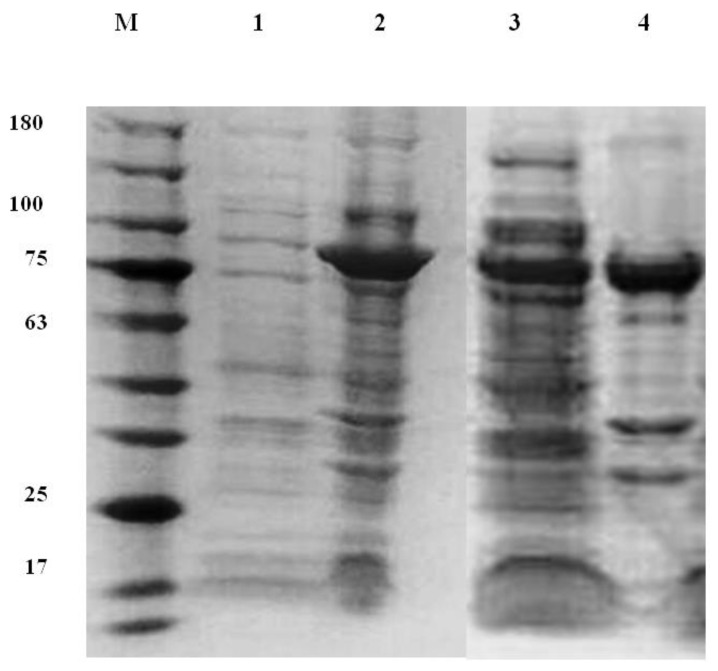
SDS-polyacrylamide gel electrophoresis of chitinase 1198 from recombinant *E. coli*. M, molecular mass; Lane 1, cell lysate of *E. coli* before IPTG (isopropylthio-β-galactoside) induction; Lane 2, cell lysate of *E. coli* after IPTG induction for 7 hours; Lane 3, supernatant; Lane 4, precipitation of *E. coli* lysate after sonication and centrifugation.

**Figure 2:**
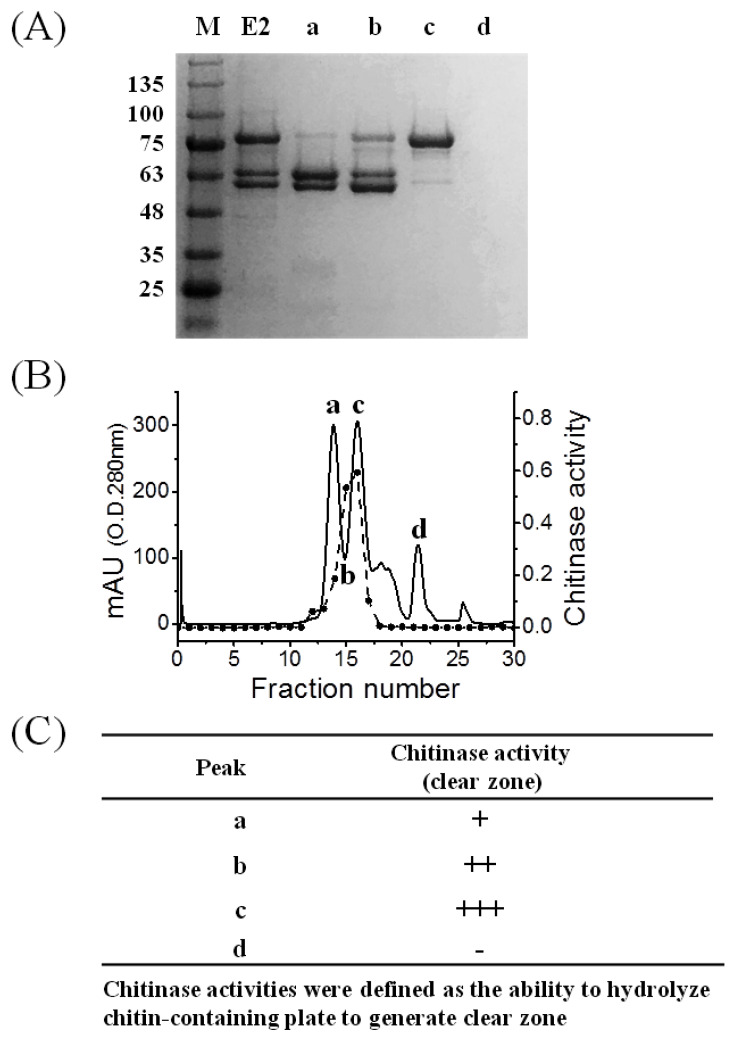
Purification of chitinase 1198 from recombinant Escherichia coli. (**A**) Lane E2, elution of chitinase 1198 from Ni-column purification. Lanes a–d, different fractions collected from gel-filtration purification. (**B**) Gel-filtration chromatography on a Superose 12 30/300 GL of E2 following Ni-column purification of chitinase 1198. Dashed line = chitinase activity of different fractions by using p-NP-(GlcNAc)2 as a substrate. (**C**) Chitinase activity from Superose 12 30/300 GL. Chitinase activity was defined as the ability to hydrolyze a chitin-containing plate to generate a clear zone. Diameters of clear zone < 1 cm, approximately 1 cm, and > 1 cm were designated as +, ++, and +++, respectively.

The CT01GL001198 gene was cloned into the pET101/D-TOPO expression vector, which produced active recombinant chitinase 1198. This protein contained a poly histidine (6 × His) tag in the C terminal of chitinase 1198 and had 693 amino acids with an expected molecular weight of 73,957.79 Da (Figure S2). This protein was detected at approximately 75 kDa in the SDS-PAGE analysis after IPTG (isopropylthio-β-galactoside) induction (Figure 1, Lane 2). Cells were disrupted through sonication and centrifugation for 20 min at 20,000× *g* to examine their protein solubility. Results indicated that approximately 40% and 60% of overexpressed chitinase 1198 were found in the supernatant (Figure 1, Lane 3) and inclusion body (Figure 1, Lane 4), respectively. The supernatant was absorbed on an Ni column, followed by purification through gel-filtration chromatography on a Superose 12 30/300 GL. Then, eluted fractions were analyzed using SDS-PAGE, and results are presented in Figure 2A. After gel-filtration chromatography purification, the peak C (Figure 2A, Lane C) appeared as a single band of approximately 75 kDa. The assay of chitinase activity with p-NP-(GlcNAc)2 as the substrate confirmed that chitinase 1198 is a chitinase (dashed line in Figure 2B). Lastly, chitinase activity was also confirmed by the ability to hydrolyze a chitin-containing plate to generate a clear zone (Figure 2C). 

### 3.2. Effects of pH and Temperature on Chitinase 1198 Activity

**Figure 3:**
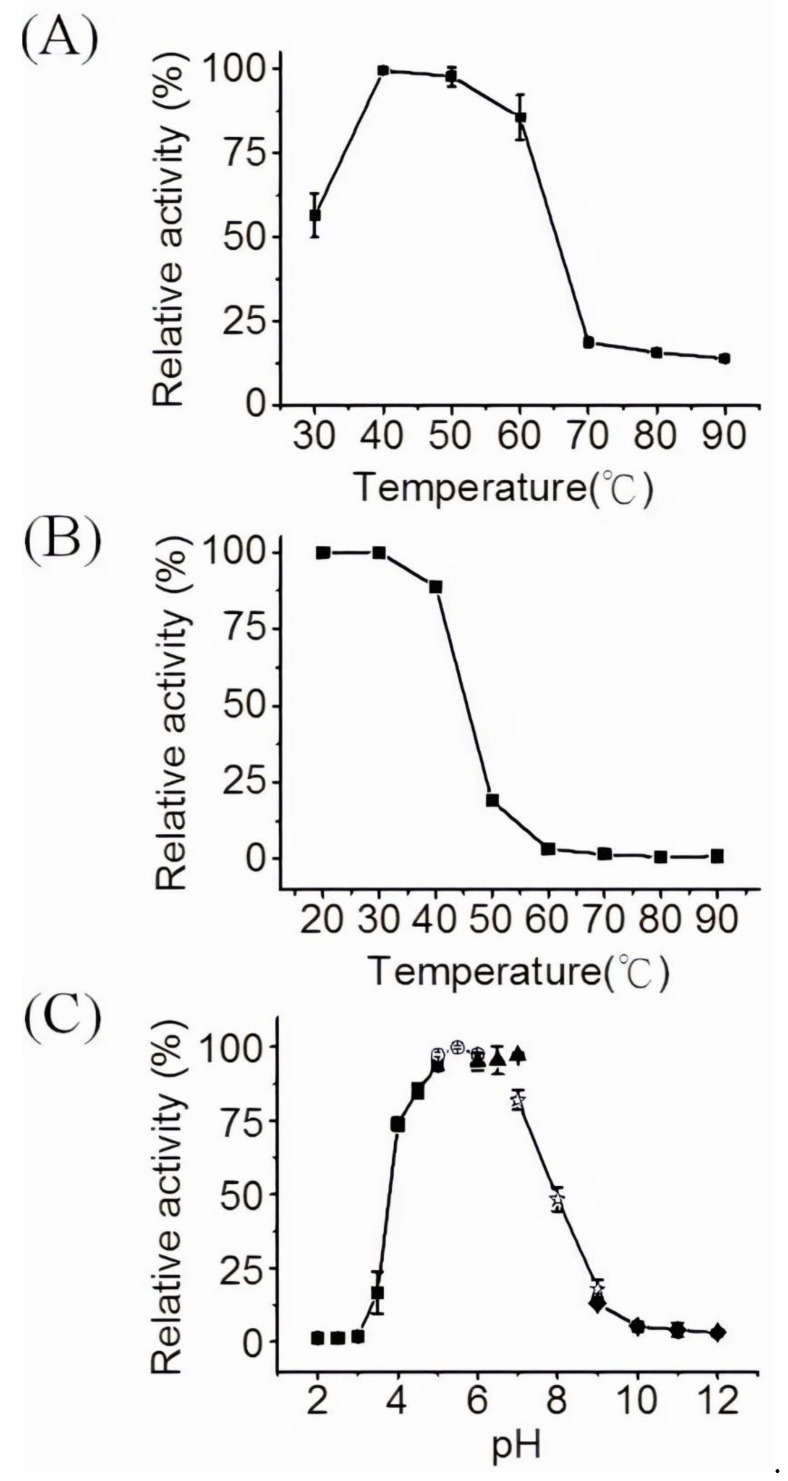
Optimization of chitinase 1198 activity by using p-NP-(GlcNAc)2 as a substrate. (**A**) Effects of temperature on chitinase 1198 activity. Chitinase activity was determined at different temperatures (30 to 90 °C). (**B**) Thermostability of chitinase 1198 was determined at different temperatures (20 to 90 °C). (**C**) Effects of pH on chitinase 1198 activity. Chitinase activity was determined under different pH conditions. Symbols: black squares = sodium citrate (pH = 2.0–5.0); open circles = sodium phosphate (pH = 5.0–6.0); black triangles = MES (pH = 6.0–7.0); open stars = Tris-HCl (pH = 7.0–9.0); black diamonds = glycine-NaOH (pH = 9.0–12.0).

The optimal temperature for chitinase 1198 activity was determined to be approximately 40 °C by using p-NP-(GlcNAc)_2_ as a substrate (Figure 3A). Chitinase 1198 retained more than 75% of its optimal enzyme activity between 40 and 60 °C. The thermostability of chitinase 1198 was determined through preincubation at different temperatures for 1 h. Chitinase 1198 retained more than 75% of its optimal activity after preincubation between 20 and 40 °C for 1 h. The activity dramatically decreased to approximately 20% at 50 °C, indicating that chitinase 1198 is temperature sensitive (Figure 3B). Chitinase 1198 activity was optimal at a pH of approximately 6.0, and it retained more than 75% and 50% of its optimal activity at a pH of 4.0–7.0 and 4.0–8.0, respectively (Figure 3C). 

### 3.3. Effect of Metal Ions on Chitinase 1198 Activity

We observed a significant increase (approximately 4%–7%) in chitinase activity through the addition of 10 mM K^+^, Ba^2+^, EDTA, Mn^2+^, and Mg^2+^, whereas other ions had a drastic inhibitory effect on enzyme activity, which can be expressed as follows: Cu^2+^ > Zn^2+^ > Co^2+^ (Table 1). Several authors have noted chitinase activation by Ca^2+^, Mg^2+^, Mn^2+^, Na^+^, and K^+^. Notably, 10 mM EDTA enhanced chitinase 1198 activity in the present study, whereas it inhibited chitinase activity by 20%–35% in other studies [39,40].

### 3.4. Activity of Chitinase 1198 on Colloidal Chitin and pNP-(GlcNAc)_1–3_ Substrates 

**Figure 4:**
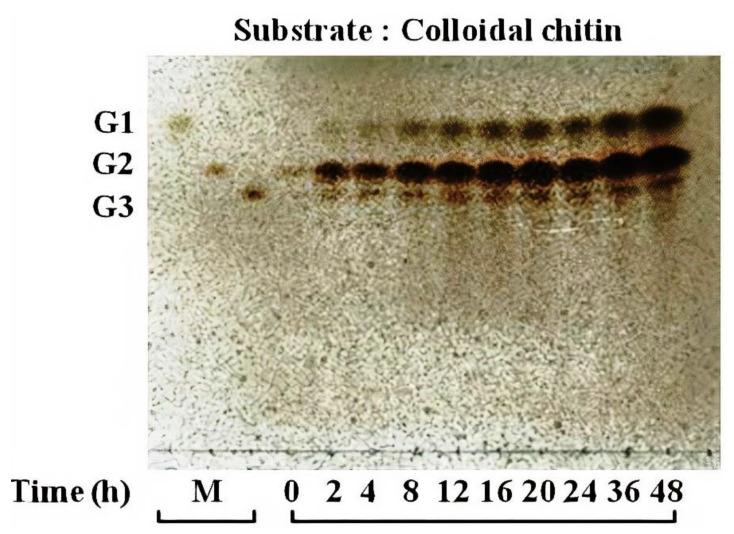
Thin-layer chromatography (TLC) analysis of reaction products of colloidal chitin incubated with chitinase 1198. Reaction mixtures containing 16.45 mg/mL of colloidal chitin as a substrate in 50 mM sodium phosphate buffer (pH = 6.5) were incubated with chitinase 1198 (3.97 M) at 40 °C for the indicated times (0, 2, 4, 8, 12, 16, 20, 24, 36, and 48 h). Lane M, standard GlcNAc or its oligomers ranging from (GlcNAc)_1_ to (GlcNAc)_3_ designated as G1 to G3.

**Figure 5:**
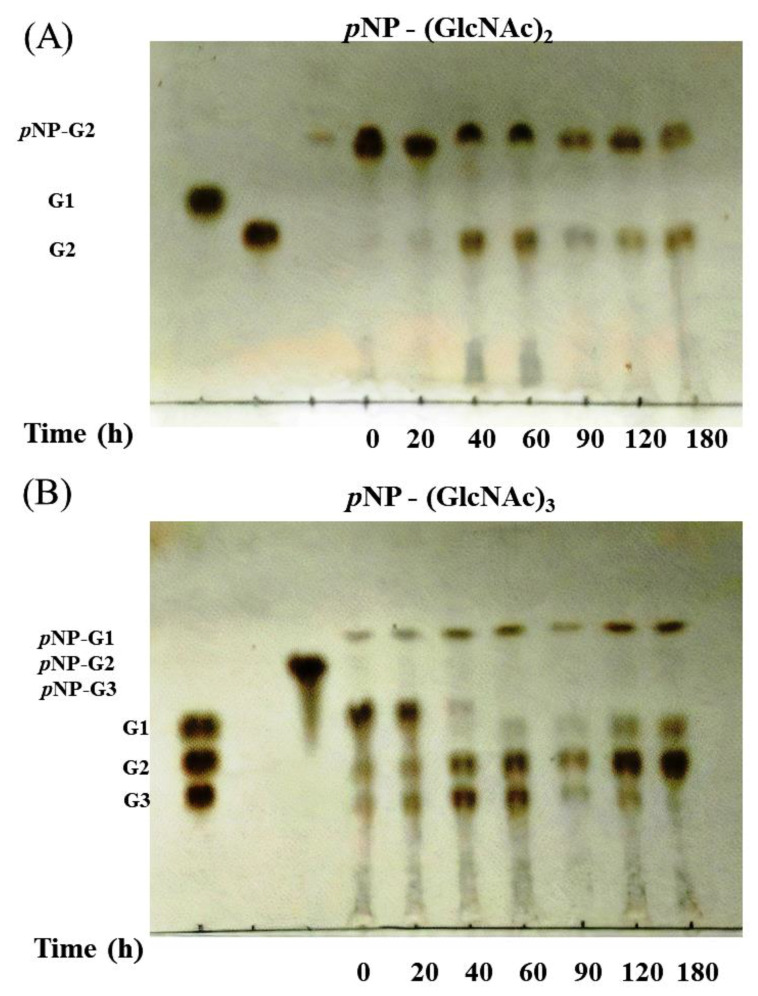
TLC analysis of reaction products of various p-nitrophenyl GlcNAc oligomers incubated with chitinase 1198. Reaction mixtures containing (**A**) 0.5 mg/mL of p-NP-(GlcNAc) or (**B**) p-NP(GlcNAc)2 as a substrate in 50 mM sodium phosphate buffer (pH = 6.5) were incubated with chitinase 1198 (0.28 M) at 40 °C for the indicated times (0, 20, 40, 60, 90, 120, and 180 min). Lane M, standard GlcNAc oligomers ranging from (GlcNAc)1 to (GlcNAc)3 (G1 to G3).

**Figure 6:**
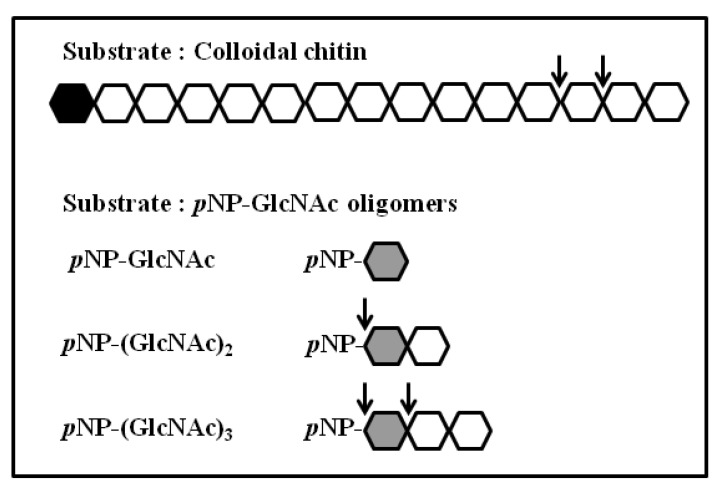
Schematic of the reaction catalyzed by chitinase 1198. Individual GlcNAc monomers are represented by hexagons. Black hexagons indicate the reducing ends of GlcNAc oligomers; gray hexagons indicate the reducing ends of pNP-GlcNAc oligomers. The arrows indicate cleavage sites in the experiments.

To determine whether chitinase 1198 proteins are endochitinase or exochitinase enzymes that recognize the ends of (GlcNAc)n chains, we next examined products generated from colloidal chitin by using TLC. In addition to small amounts of (GlcNAc)_1_ and (GlcNAc)_3_, the main product formed was (GlcNAc)_2_, indicating that these proteins recognized the ends of chitin oligomers and are exochitinase enzymes (Figure 4). Our results indicated that the concentrations of (GlcNAc)_1_ and (GlcNAc)_2_ increased gradually up to 48 h of incubation, whereas that of (GlcNAc)_3_ leveled off after 8 h. The ratio of (GlcNAc)_2_ to (GlcNAc)_1_ at the end of the reaction was approximately 3:1. Chitin conversion expressed as the amount of (GlcNAc)_2_ produced was 60% after 48 h. To determine whether chitinase 1198 is an endo- or exotype enzyme, we examined the enzyme activity toward pNP-(GlcNAc)_1–3_ substrates. Chitinase 1198 did not hydrolyze pNP-GlcNAc (Table 2). In contrast, pNP-(GlcNAc)_2_ and pNP-(GlcNAc)_3_ were suitable substrates for chitinase 1198. Thus, chitinase 1198 is predicted to function as an endochitinase. To identify the end (reducing or nonreducing) recognized by chitinase 1198, pNP-(GlcNAc)_2_ and pNP-(GlcNAc)_3_ products obtained following chitinase 1198 digestion were analyzed using TLC (Figure 5). Results revealed that pNP-(GlcNAc)_2_ was digested exclusively into pNP and (GlcNAc)_2_ (Figure 5A, pNP was detected using ultraviolet light; data not shown), and pNP-(GlcNAc)_3_ was digested into pNP, pNP-(GlcNAc)_1_, (GlcNAc)_2_, and (GlcNAc)_3_ (Lanes 6 and 7). Over a longer period, the released (GlcNAc)_3_ was further degraded into (GlcNAc)_1_ and (GlcNAc)_2_ (Lanes 9 and 10). Taken together, these results suggest that chitinase 1198 recognizes the nonreducing end of chitin and cleaves the second or third glycosidic linkage from the nonreducing end. Chitinase 1198 did not exhibit NAGase activity, which cleaves the glycosidic linkage from the nonreducing end. Instead, GlcNAc spots observed in colloidal chitin or pNP-(GlcNAc)_3_ were further digested from the intermediate product (GlcNAc)_3_ in the second glycosidic linkage from the nonreducing end (Figure 6). The kinetics parameters of chitinase were determined using pNP-(GlcNAc)_2_ as a substrate. Our kinetics parameters are shown in Table 3.

### 3.5. Molecular Modeling of Chitinase 1198 and These Enzymes with Chitin Oligomers (GlcNAc Repeat Unit: 12 and 3)

**Figure 7:**
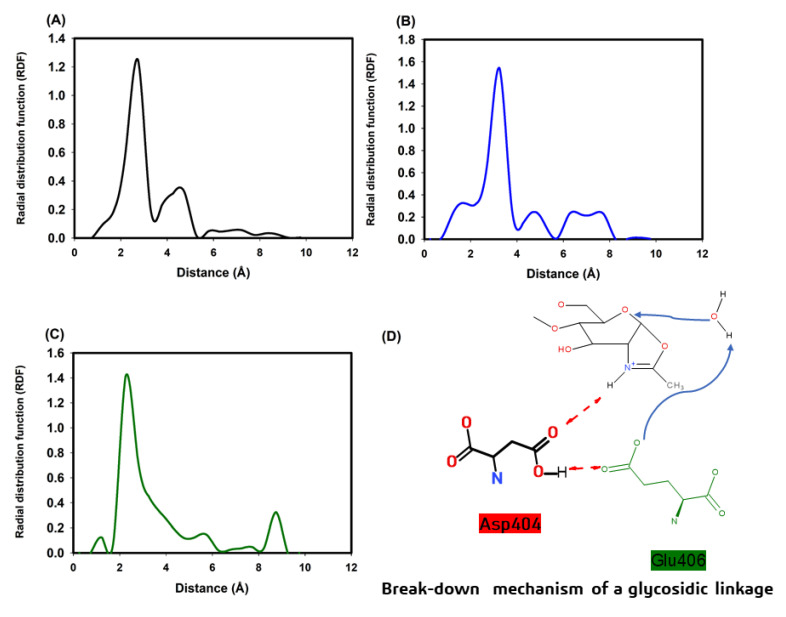
Radial distribution function of the chitinase 1198 DXXDXDXE motif and chitin oligomers. (**A**) Chitin repeat unit 12 in the Asp440 second GlcNAc repeat unit complex structures. (**B**) Chitin repeat unit 12 in the Asp440 third GlcNAc repeat unit complex structures. (**C**) Chitin repeat unit 3 in the Asp440 second GlcNAc repeat unit complex structures. (**D**) Breakdown mechanism of a glycosidic linkage [26,27].

The predicted secondary structure of chitinase 1198 is composed of 13 α-helixes, 11 η-helixes, and 15 β-sheets. The 3D structure of chitinase 1198 is illustrated in Figure S3(B). A comparison between the 3D structure of chitinase 1198 and other chitinases (Figure S4) is presented in Figure S5. Our results indicated that the 3D chitinase 1198 structures of the active site were conserved. The snapshots of chitinase 1198 with chitin oligomers (GlcNAc repeat units: 12 and 3) are displayed in Figures S6–S8. Interactions between Asp 440 (the last Asp residue in the chitinase 1198 DXXDXDXE motif) and the nitrogen atoms of chitin oligomers (GlcNAc repeat units: 12 and 3) were investigated. The RDF of the two complex structures is revealed in Figure 7. For chitinase 1198 with chitin oligomers (GlcNAc repeat unit: 12), the maximum RDF value was 2.75 Å, and the Asp440 of chitinase 1198 could form hydrogen bonds with the nitrogen atom of the second GlcNAc repeat unit from the nonreducing end. For chitinase 1198 with chitin oligomers (GlcNAc repeat unit: 12), the maximum RDF value was 2.25 Å, and the Asp440 of chitinase 1198 could form hydrogen bonds with the nitrogen atom of the third GlcNAc repeat unit from the nonreducing end. For chitinase 1198 with chitin oligomers (GlcNAc repeat unit: 3), the maximum RDF value was 3.25 Å, and the Asp440 of chitinase 1198 could form hydrogen bonds with the nitrogen atom of the second GlcNAc repeat unit from the nonreducing end. The predicted binding free energies of each chitinase 1198-chitin oligomer (GlcNAc repeat units: 12 and 3) were obtained from 100-ns NTP MD simulations and by using the SIE method, with both processes using the same parameters. All the results are listed in Table 4. For chitinase 1198 with chitin oligomers (GlcNAc repeat unit: 12), the binding free energies were 12.46 ± 0.14 and −11.76 ± 0.09 kcal/mol for the Asp440 second GlcNAc repeat unit and the Asp440 third GlcNAc repeat unit, respectively. For chitinase 1198 with chitin oligomers (GlcNAc repeat unit: 3), the binding free energy was −9.99 ± 0.11 kcal/mol for the Asp440 second GlcNAc repeat unit. The comparison between our simulated and predicted binding free energies revealed that chitinase 1198 may provide efficient binding affinities with chitin oligomers (GlcNAc repeat unit: 12), particularly in Asp440 second GlcNAc repeat unit complex structures.

## 4. Discussion

The chitinase 1198 gene encodes a protein with 693 amino acids. An analysis of the conserved domains of chitinase 1198 in the National Center For Biotechnology Information database revealed a typical GH18 chitinase (GH18_chitinase at residues 229–647) [41]. Multiple sequence alignments of the deduced protein sequence of chitinase 1198 with chitinase from *Arthobacter* (PDB ID: 1KFW), (PDB ID: 4W5U), *Bacillus circulans* (PDB ID: 1ITX), *Serratia marcescens* (PDB ID: 2WLY), and nematophagous fungus (PDB ID: 3G6L) revealed the presence of conserved active domains in chitinase 1198 (Figure S4). In total, 44 conserved amino acids and 1 conserved active-site motif consisting of aspartate (D) and glutamate (E) residues forming a DXXDXDXE motif were found in chitinase 1198 [27]. The predicted 3D structure of chitinase 1198 was compared with that of other chitinases. The 3D structures of the active site were conserved (Figure S5). The n-terminal of chitinase 1198 (residues 1–201) contains many disordered structures lacking intermolecular hydrogen bonding force for stabilizing the structure; therefore, it is susceptible to proteolysis. This finding is consistent with the mass spectrophotometer analysis results of two major bands in Lanes a and b with an MW of approximately 63 kD (Figure 2A). 

In family 18 chitinase reactions, the last Asp residue of the DXXDXDXE motif can form hydrogen bonds with the nitrogen atom of the chitin unit from the nonreducing end; subsequently, the Glu residue can break down glycosidic bonds in chitin oligomers (Figure 7D) [2,27,42,43,44]. Thus, the intermolecular hydrogen bond of the Asp residue with the nitrogen atom of the chitin unit is essential for chitin degradation. Our RDF profiles revealed that Asp440 (the last Asp residue of the DXXDXDXE motif) can form hydrogen bonds with the nitrogen atoms of the chitin repeat unit in the three simulation cases. Then, the predicted binding free energies are as follows: the Asp440 GlcNAc second repeat unit (GlcNAc repeat unit: 12) > Asp440 third GlcNAc repeat unit (GlcNAc repeat unit: 12) > Asp440 second GlcNAc repeat unit (GlcNAc repeat unit: 3). Our experimental results also revealed that the main product formed was (GlcNAc)_2_, followed by small amounts of (GlcNAc)_1_ and (GlcNAc)_3_, when chitinase 1198 reacted with colloidal chitin oligomers (Figure 4). Therefore, our experimental data were consistent with our simulations.

To sum up, our predicted and experimental results revealed that chitinase 1198 is more likely to bind chitin oligomers with the main hydrogen bonds of the Asp440 second GlcNAc repeat unit and degrade chitin oligomers to (GlcNAc)_2_ molecules. Moreover, chitinase 1198 is less likely to bind chitin oligomers with the main hydrogen bonds of the Asp440 third GlcNAc repeat unit and degrade chitin oligomers to (GlcNAc)_3_ molecules. Lastly, chitinase 1198 can bind (GlcNAc)_3_ molecules with the main hydrogen bonds of the Asp440 second GlcNAc repeat unit and degrade chitin oligomers to (GlcNAc)_2_ molecules.

## 5. Conclusions

In this study, we used molecular simulations and experimental methods to study chitinase 1198 enzymes. Our predicted binding free energies were as follows: the Asp440 second GlcNAc repeat unit (GlcNAc repeat unit: 12) > Asp440 third GlcNAc repeat unit (GlcNAc repeat unit: 12) > Asp440 second GlcNAc repeat unit (GlcNAc repeat unit: 3). Our experimental results revealed that the main product formed was (GlcNAc)_2_, followed by small amounts of (GlcNAc)_1_ and (GlcNAc)_3_, when chitinase 1198 reacted with colloidal chitin oligomers. Therefore, our experimental data were consistent with our simulations.

Our results suggest that chitinase 1198 recognizes the nonreducing end of chitin and cleaves the second or third glycosidic linkage from the nonreducing end. Our preliminary findings indicate that chitinase 1198 is a novel endochitinase that is worthy of further investigation for the commercial production of chitin oligosaccharides; moreover, this enzyme can hydrolyze chitin oligomers to (GlcNAc)_1–3_.

## Figures and Tables

**Table 1:** Effect of metal ions on chitinase 1198 activity.

Metal Ions	Relative Activity (%)
K^+^ (KCl)	106.4 ± 0.1
Na^+^ (NaCl)	101.6 ± 1.9
Ba^2+^ (BaCl2)	106.3 ± 0.2
Ca^2+^ (CaCl2)	101.6 ± 1.2
Co^2+^ (CoCl2)	81.9 ± 0.8
EDTA	106.3 ± 3.2
Cu^2+^ (CuSO4)	36.1 ± 1.6
Mn^2+^ (MnSO4)	106.4 ± 0.1
Mg^2+^ (MgSO4)	104.7 ± 0.1
Zn^2+^ (ZnSO4)	65.3 ± 1.1

**Table 2:** Hydrolytic activity of chitinase 1198 on pNP-(GlcNAc)_1–3_.

Substrate	Specific Activity * (Units/mg)	Relative Activity (%)
pNP-GlcNAc	0	0
pNP-(GlcNAc)_2_	3.09 × 10^5^	91.4
pNP-(GlcNAc)_3_	3.38 × 10^5^	100

* One unit of chitinase activity is defined as the release of 1 μmoL p-nitrophenol/min.

**Table 3:** Comparison of kinetic parameters of different chitinase 1198 constructs on pNP-(GlcNAc)_2_.

Chitinase1198	*k*_cat_ (s^−1^)	*K*_M_ (μM)	*k*_cat_/*K*_M_ (s^−1^μM^−1^)
Wild type (WT)	1.91 ± 0.33	170.40 ± 27.65	11.43 × 10^−3^
Truncated	0.36 ± 0.04	58.30 ± 11.01	6.38 × 10^−3^

**Table 4:** Detailed information of solvated interaction energy free energy calculations for chitinase 1198.

Number of Peptide Inhibitors	Chitinase 1198 with the Chitin Oligomers (GlcNAc Repeat Unit: 12) (the Asp440-2nd GlcNAc Repeat Unit)	Chitinase 1198 with the Chitin Oligomers (GlcNAc Repeat Unit: 12) (the Asp440-3rd GlcNAc Repeat Unit)	Chitinase 1198 with the Chitin Oligomers (GlcNAc Repeat Unit: 3) (the Asp440-2nd GlcNAc Repeat Unit)
Inter vdW	−92.36 ± 1.20	−90.26 ± 0.67	−66.44 ± 0.82
Inter Coulomb	−23.33 ± 0.56	−36.72 ± 0.87	−23.91 ± 0.53
Reaction Field	39.14 ± 0.53	58.26 ± 0.69	33.64 ± 0.38
Cavity	−14.81 ± 0.19	−15.98 ± 0.73	−11.09 ± 0.06
Constant	−2.89	−2.89	−2.89
△G_Binding_ (kcal/mol)	−12.46 ± 0.14	−11.76 ± 0.09	−9.99 ± 0.11

## Supplementary Materials

The following are available online at https://www.mdpi.com/2073-4360/12/8/1648/s1, Figure S1. The phylogenetic tree with the 12 chitinase-encoding genes sequences of Chitinibacter tainanensis CT01 and the other chitinase genes sequences. Figure S2 Amino acid and DNA sequence of chitinase 1198. Figure S3. (A) Secondary structures of chitinase 1198. (B) Predicted 3D structures of chitinase 1198. Figure S4. Multiple sequence alignment of the active site (green box: DXXDXDXE one letter amino acid motif) of the deduced protein sequence of chitinase 1198 with chitinase from Arthobacter (PDB ID: 1KFW), (PDB ID: 4W5U), Bacillus circulans (PDB ID: 1ITX), Serratia marcescens (PDB ID:2WLY), and chitinase from nematophagous fungus (PDB ID: 3G6L). Figure S5. Comparing the Chi1198 3D structures (colored in green) with chitinases from Arthobacter (PDB ID: 1KFW and colored in purple), (PDB ID: 4W5U and colored in gray), Bacillus circulans (PDB ID: 1ITX and colored in cyan), Serratia marcescens (PDB ID:2WLY and colored in tints), and chitinase from nematophagous fungus (PDB ID: 3G6L and colored in blue). Figure S6. Chi1198 with chitin oligomers (repeat unit: 12) and Family 18 chitinase: DXXDXDXE amino acid motif. (The distance between the Asp440 of chitinase1198 and the nitrogen atom of the 2nd repeat unit from the nonreducing end). Figure S7. Chi1198 with chitin oligomers (repeat unit: 12) and Family 18 chitinase: DXXDXDXE amino acid motif. (The distance between the Asp440 of chitinase1198 and the nitrogen atom of the 3rd repeat unit from the nonreducing end). Figure S8. Chi1198 with chitin oligomers (repeat unit: 3) and Family 18 chitinase: DXXDXDXE amino acid motif. (The distance between the Asp440 of chitinase1198 and the nitrogen atom of the 2nd repeat unit from the nonreducing end).
