# Supplementary material for: Gene Cloning, Characterization, and Molecular Simulations of a Novel Recombinant Chitinase from Chitinibacter Tainanensis CT01 Appropriate for Chitin Enzymatic Hydrolysis
Source: Polymers (Basel). 2020 Jul 24;12(8):1648. doi: 10.3390/polym12081648 (PMC7463862; doi:10.3390/polym12081648)
Supplement: Supplementary file 1 [file polymers-12-01648-s001.pdf]

polymers

MDPI

Gene cloning, characterization, and molecular simulations of a novel recombinant chitinase from Chitinibacter tainanensis CT01 appropriate for chitin enzymatic hydrolysis

Yeng-Tseng Wang[1,2,3,4 and 5*] and Po-Long Wu[6]

[1] Department of Biochemistry, College of Medicine, Kaohsiung Medical University, Kaohsiung City 80708, Taiwan, ROC
[2] Drug Development and Value Creation Research Center, Kaohsiung Medical University, Kaohsiung, Taiwan, ROC
[3.] Graduate Institute of Medicine, Kaohsiung Medical University, Kaohsiung, Taiwan, ROC
[4] Department of Medical Research, Kaohsiung Medical University Hospital, Kaohsiung, Taiwan, ROC
[5] School of Post-Baccalaureate Medicine, College of Medicine, Kaohsiung Medical University, Taiwan
[6] Biotech Business Center, Refining & Manufaturing Research Institute, CPC Corporation, Chiayi City 60051, Taiwan, ROC. 078760@cpc.com.tw
* Correspondence: c00jsw00@kmu.edu.tw or c00jsw00@gmail.com; Tel.: +886- 07-3121101

Received: 07 June 2020; Accepted: 20 July 2020; Published: date

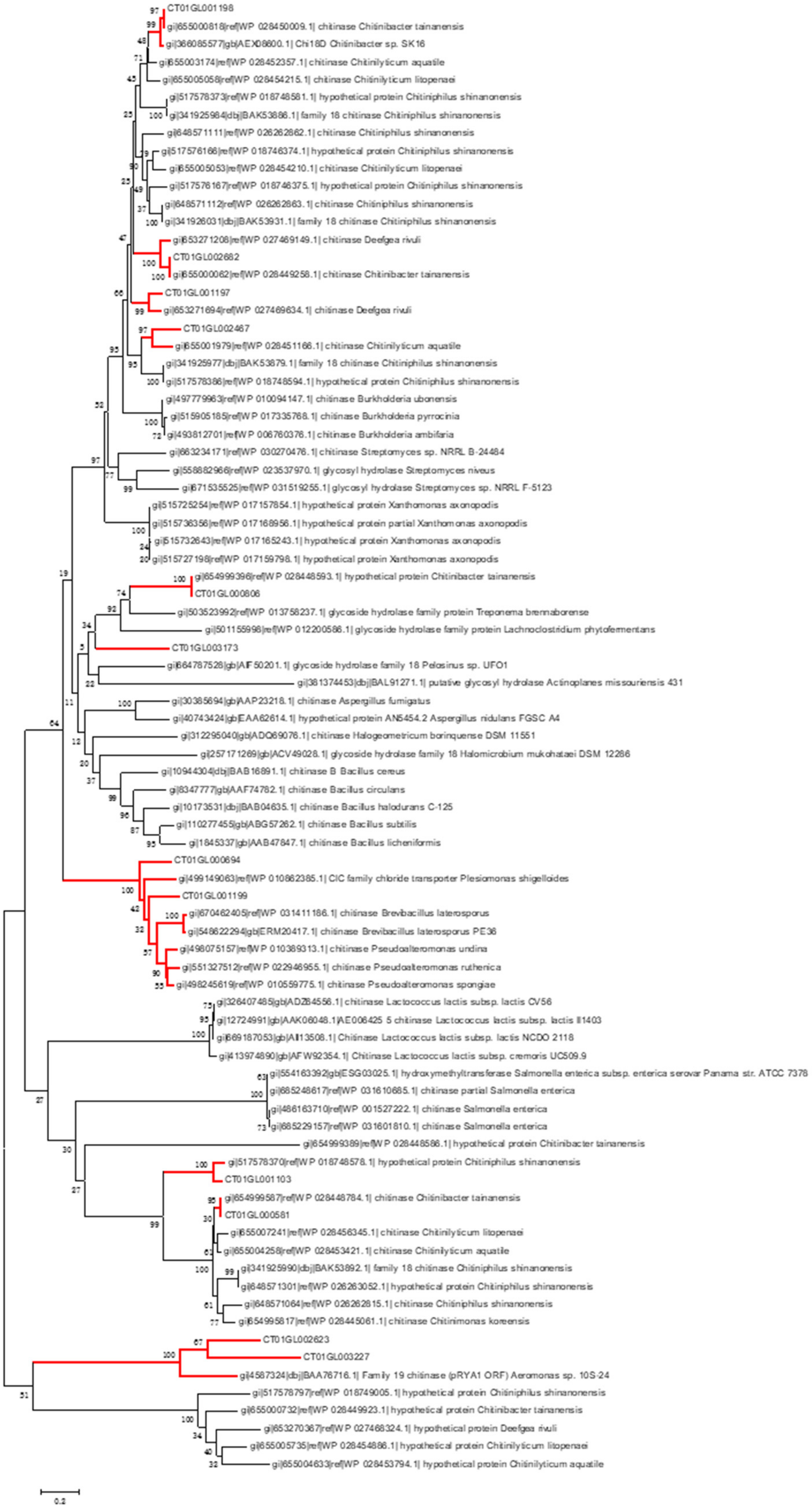

Figure 1. The phylogenetic tree with the 12 chitinase-encoding genes sequences of Chitinibacter tainanensis CT01 and the other chitinase genes sequences.

```
atgtcgcactttaatcgttttgctgtagctgctattcctgctgcactgatggccgtggccagcttctcttacgctgctgatgcgtggaaagaaggt
M S H F N R F A V A A I P A A L M A V A S F S Y A A D A W K E G
tcgacctataacgcaggcactgttgtttcatatgccggtaacgactaccaagctctggtgactcacactgcctacgtgggcgctaactggaaccct
S T Y N A G T V V S Y A G N D Y Q A L V T H T A Y V G A N W N P
gcttcaaccccaacactgtggaaactggttgctaccggttctgctacgccagcgccaaccgcgacgccagtaacaacggctaagccaaccacggcc
A S T P T L W K L V A T G S A T P A P T A T P V T T A K P T T A
ccaacaactgcgccaaccaccgcaccgacaactgctccaactaccgcgccaactactggcccagtagcaggttgtggctctgtagcggcttggaat
P T T A P T T A P T T A P T T A P T T G P V A G C G S V A A W N
tcaactgctgcgtatagcggcggcgcagtagttgcctacaacggcggcaaatactcagctaaatggtggacacaaggtcaggcgccttctgcaact
S T A A Y S G G A V V A Y N G G K Y S A K W W T Q G Q A P S A T
gatcagtggggcccatggaaatacgaaggcgagtgcggcccagctgtaactgcgacgccaactgtagcgccaacaccaaccccagttggcatgacc
D Q W G P W K Y E G E C G P A V T A T P T V A P T P T P V G M T
ccagcaccaaccgtagcgccaactccaactgcagttccaactgcagcgccaacaccagttgctaccttggctccaggtcaagaagtgcctccacct
P A P T V A P T P T A V P T A A P T P V A T L A P G Q E V P P P
gcgcaagcgcaagttggctcttacttcactcagtggggcgtgtatggtcgtgactaccaagttgccgacatcatctctagcggcgctgctcagcaa
A Q A Q V G S Y F T Q W G V Y G R D Y Q V A D I I S S G A A Q Q
ctgaccttcatcaactacgccttcggtaacatctaccagaaaaatggtggttacgagtgcggtatcgtgaacaaactggaaccaggtgcaactgat
L T F I N Y A F G N I Y Q K N G G Y E C G I V N K L E P G A T D
gcaaatgcaccaggcgctggtactggtggcgatgcttgggccgactttggtctgactgcgaaacgtcgtgttgatccagctgatcaaatcaagtgg
A N A P G A G T G G D A W A D F G L T A K R R V D P A D Q I K W
gacgacaaactggccggtaacttccgtgagttccaggcttataagaaaaaattcccagacaccaaactgttcatctctctgggtggctggacttgg
D D K L A G N F R E F Q A Y K K K F P D T K L F I S L G G W T W
tcgaaatggttctctgctgcatcgaaaaccgacgcgctgcgtaaacagctggttaaatcgtgtatcgacatctacatcaaaggtaacttgccagtg
S K W F S A A S K T D A L R K Q L V K S C I D I Y I K G N L P V
gtggatggccgtggtggcgcaggttcagctgcgaacatcttcgatggtatcgatatcgactgggaattcccaggtgttcaaggcgtgggttacaac
V D G R G G A G S A A N I F D G I D I D W E F P G V Q G V G Y N
actgtagcgcctgaagacaaacaaaacttcaccctgttgctggctgaattccgcaaacaactggatgaactggctgccgctaaccagaagaaatac
T V A P E D K Q N F T L L L A E F R K Q L D E L A A A N Q K K Y
tacctgaccgttgctatcggtgtaggtcgtgacaagatcgaaatgactgagccacgcgagtacgcacgttacctcgactggatcaacatgatgact
Y L T V A I G V G R D K I E M T E P R E Y A R Y L D W I N M M T
tacgactacaacggcggctggaacgcacaaggcccgactgacttccagtctcacctgttcgctgatccaagcaacccacagtacaagtgctctggc
Y D Y N G G W N A Q G P T D F Q S H L F A D P S N P Q Y K C S G
aaaccagccgacaagtgctacggtgaccgcagcctggtgtcttactacaacaccgatgacgcagtgaacctgctgatccaagctggcgtgaatccg
K P A D K C Y G D R S L V S Y Y N T D D A V N L L I Q A G V N P
aagaaactggtggttggtattcctaagtacggtcgtggctggactggtgtgaccaatgtgaacaacggtctgtaccagaaagcgactgacgcagca
K K L V V G I P K Y G R G W T G V T N V N N G L Y Q K A T D A A
cgcggtacctacgagaaaggcatcgaagacttcaaagtactgaaaaacgctgcgggtactgtgtatgtacacccagtaactaaacagtcttacaag
R G T Y E K G I E D F K V L K N A A G T V Y V H P V T K Q S Y K
ttcgacggttctactttctggtcgtacgatacacctgaagtgatccaaactaagatcgactacgccaaagccaaagggctgaacggtggtgttc
F D G S T F W S Y D T P E V I Q T K I D Y A K A K G L N G G V F
agctggtcactggatggtgacgacagcgcagcaacactgtcgaaagccatgggtaaagcccgtcagaagggcgagctcaattcgaagcttgaaggt
S W S L D G D D S A A T L S K A M G K A R Q K G E L N S K L E G
aagcctatccctaaccctctcctcggtctcgattctacgcgtaccggtcatcatcaccatcac
K P I P N P L L G L D S T R T G H H H H H
```

Figure 2. Amino acid and DNA sequence of chitinase 1198.

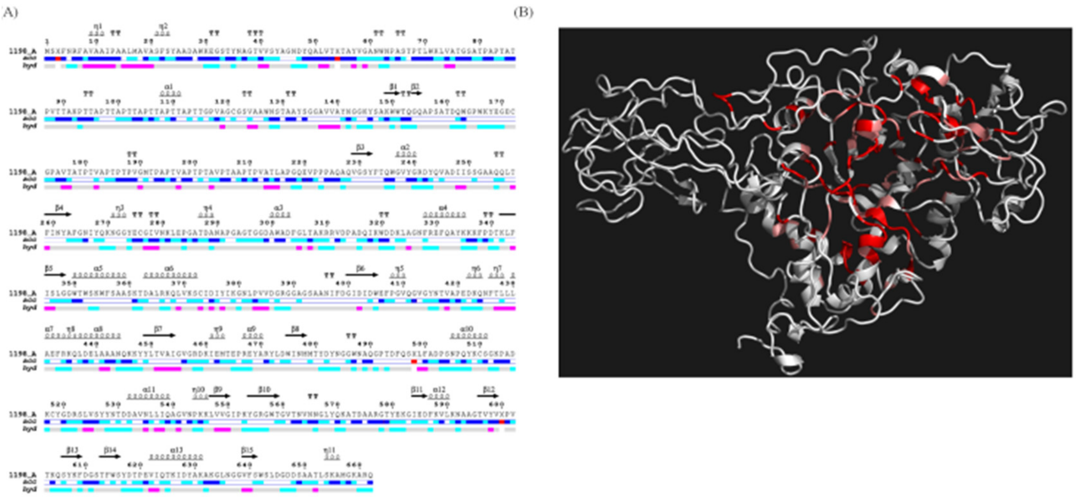

Figure 3. (**A**) Secondary structures of chitinase 1198. (**B**) Predicted 3D structures of chitinase 1198.

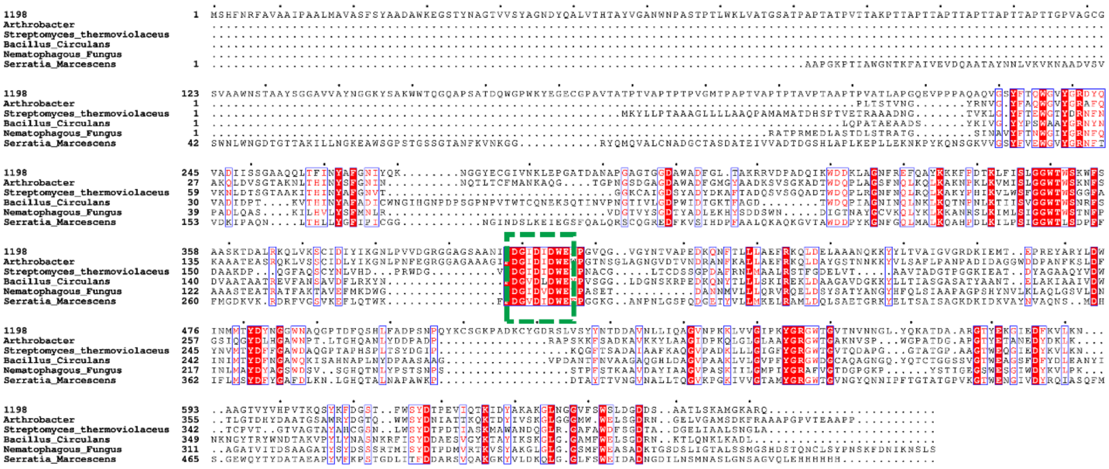

Figure 4. Multiple sequence alignment of the active site (green box: DXXDXDXE one letter amino acid motif) of the deduced protein sequence of chitinase 1198 with chitinase from Arthobacter (PDB ID: 1KFW), (PDB ID: 4W5U), Bacillus circulans (PDB ID: 1ITX), Serratia marcescens (PDB ID:2WLY) and chitinase from nematophagous fungus (PDB ID: 3G6L).

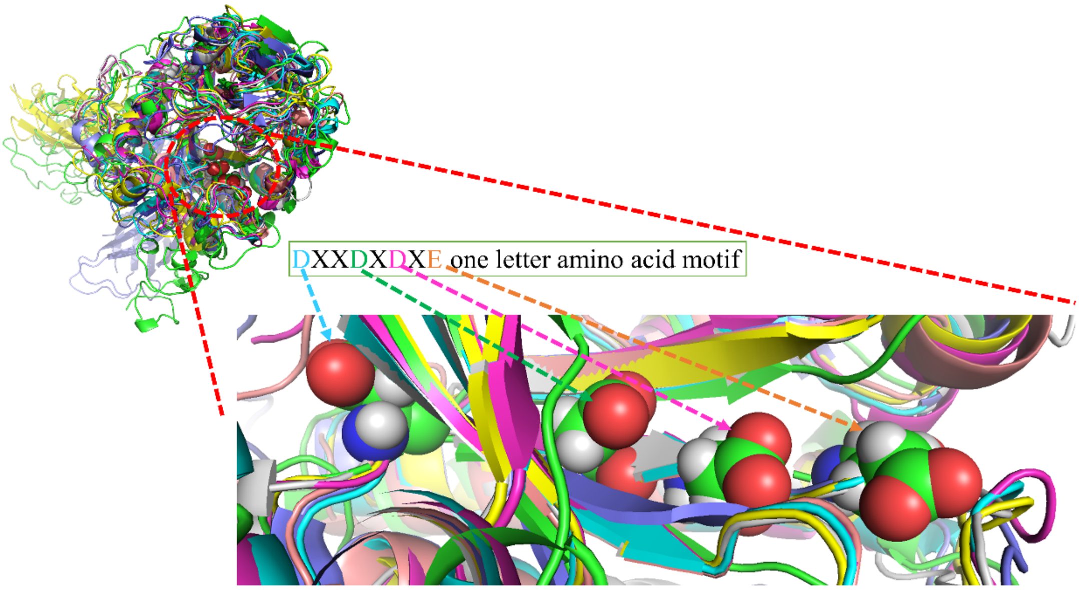

Figure 5. Comparing the Chi1198 3D structures (colored in green) with chitinases. from Arthobacter (PDB ID: 1KFW and colored in purple), (PDB ID: 4W5U and colored in gray), Bacillus circulans (PDB ID: 1ITX and colored in cyan), Serratia marcescens (PDB ID:2WLY and colored in tints) and chitinase from nematophagous fungus (PDB ID: 3G6L and colored in blue).

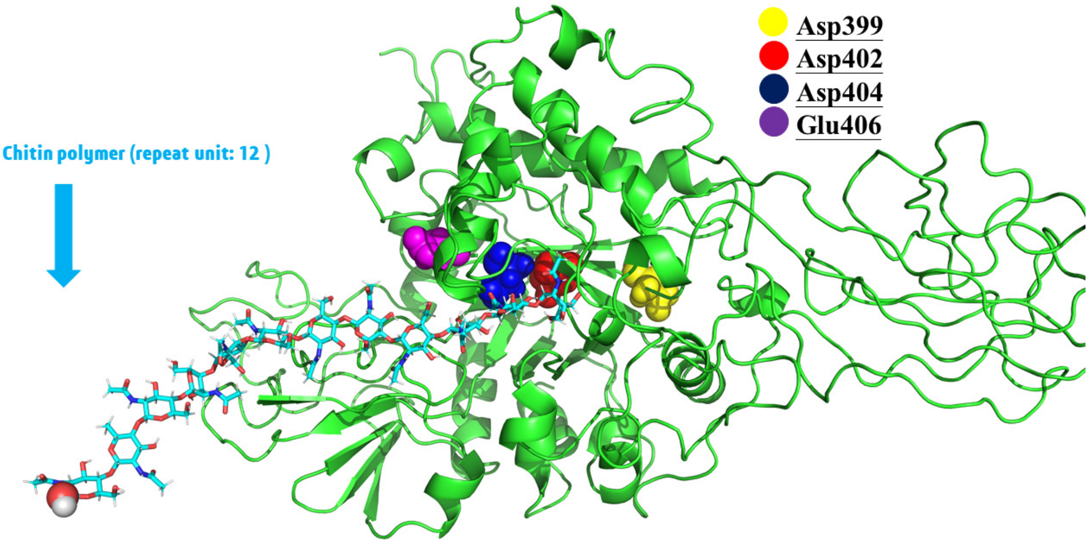

Figure 6. Chi1198 with chitin oligomers (repeat unit: 12) and Family 18 chitinase: DXXDXDXE amino acid motif. (The distance between the Asp440 of chitinase1198 and the nitrogen atom of the 2nd repeat unit from the nonreducing end).

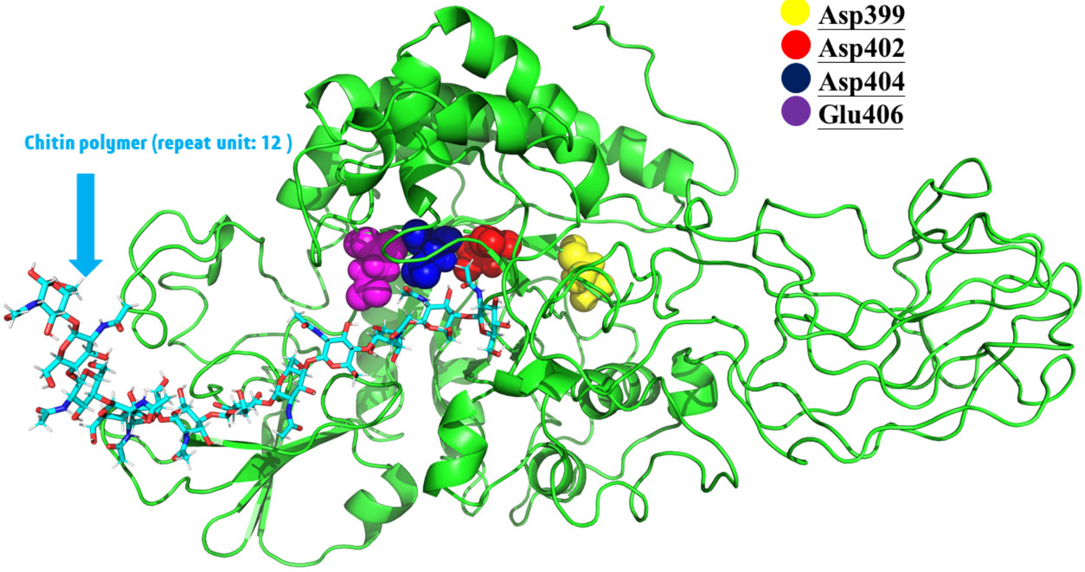

Figure 7. Chi1198 with chitin oligomers (repeat unit: 12) and Family 18 chitinase: DXXDXDXE amino acid motif. (The distance between the Asp440 of chitinase1198 and the nitrogen atom of the 3rd repeat unit from the nonreducing end).

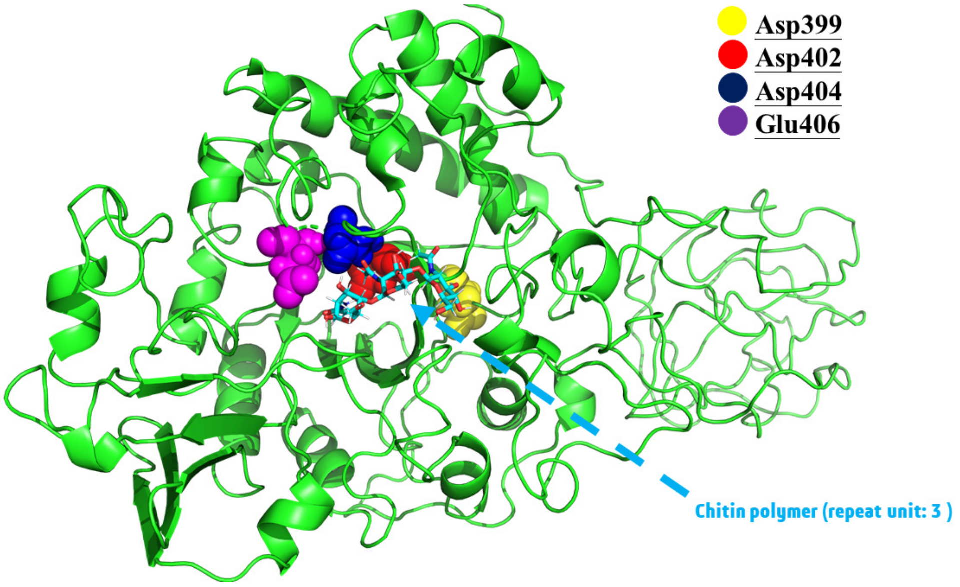

Figure 8. Chi1198 with chitin oligomers (repeat unit: 3) and Family 18 chitinase: DXXDXDXE amino acid motif. (The distance between the Asp440 of chitinase1198 and the nitrogen atom of the 2nd repeat unit from the nonreducing end).

© 2018 by the authors. Submitted for possible open access publication under the terms and conditions of the Creative Commons Attribution (CC BY) license (http://creativecommons.org/licenses/by/4.0/).
